# Cost-utility analysis of infliximab and adalimumab for refractory ulcerative colitis

**DOI:** 10.1186/1478-7547-7-20

**Published:** 2009-12-11

**Authors:** Feng Xie, Gord Blackhouse, Nazila Assasi, Kathryn Gaebel, Diana Robertson, Ron Goeree

**Affiliations:** 1 Department of Clinical Epidemiology and Biostatistics, McMaster University, Hamilton, Ontario, Canada; 2 Programs for Assessment of Technology in Health (PATH) Research Institute, St. Joseph's Healthcare, Hamilton, Ontario, Canada

## Abstract

**Objective:**

To evaluate cost-utility of infliximab and adalimumab for the treatment of moderate-to-severe ulcerative colitis (UC) refractory to conventional therapies in Canada.

**Methods:**

A Markov model was constructed to evaluate incremental cost-utility ratios (ICUR) of 5 mg/kg and 10 mg/kg infliximab and adalimumab therapies compared to 'usual care' in treating a hypothetical cohort of patients (aged 40 years and weighing 80 kg) over a five-year time horizon from the perspective of a publicly-funded health care system. Clinical parameters were derived from the Active Ulcerative Colitis Trials 1 and 2. Costs were obtained through provincial drug benefit plans. ICUR was the main outcome measure and both deterministic and probabilistic sensitivity analyses were conducted.

**Results:**

Compared to the strategy A ('usual care') in the base case analysis, the ICURs were CA$358,088/QALY for the strategy B ('5 mg/kg infliximab + adalimumab') and CA$575,540/QALY for the strategy C ('5 mg/kg and 10 mg/kg infliximab + adalimumab'). The results were sensitive to: the remission rates maintained in responders to 'usual care' and to 5 mg/kg infliximab, the rate of remission induced by adalimumab in non-responders to 5 mg/kg infliximab, early surgery rate, and utility values. When the willingness to pay (WTP) was less than CA$150,000/QALY, the probability of 'usual care' being the optimal strategy was 1.0. The probability of strategy B being optimal was 0.5 when the WTP approximated CA$400,000/QALY.

**Conclusions:**

The ICURs of anti-TNF-α drugs were not satisfactory in treating patients with moderate-to-severe refractory UC. Future research could be aimed at the long-term clinical benefits of these drugs, especially adalimumab for patients intolerant or unresponsive to infliximab treatment.

## Introduction

Ulcerative colitis (UC) is a chronic inflammatory disease of the gastrointestinal tract of unknown etiology [1]. It is characterized by diffuse mucosal inflammation limited to the colon. The most consistent clinical manifestation of UC is the presence of blood and mucus mixed with stool, accompanied by lower abdominal cramping which is most intense during bowel movements. Patients with refractory UC present persistent acute symptoms despite anti-inflammatory therapies or have chronically active disease requiring continuous treatment and long-term follow-up [2]. Conventional medical management of active UC includes: 5-aminosalicylates (5-ASA), corticosteroids, and immunosuppressants [3]. The therapeutic approach is determined by the severity of the symptoms and the degree of intestinal involvement. Surgical management may be necessary in poorly controlled or recurrent UC.

The introduction of novel biological therapies has changed the therapeutic approach to UC, particularly in patients with severe and refractory disease. Tumor necrosis factor α (TNF-α) is found in increased concentrations in blood, colonic tissue, and stools of patients with UC [4]. Infliximab (Remicade^®^, Schering) and adalimumab (Humira^®^, Abbott) are recombinant monoclonal antibodies that bind to human TNF-α, neutralizing its biologic activity [5-7]. Infliximab has been demonstrated to induce and maintain clinical response and remission in patients with moderate-to-severe UC who have not responsed to conventional therapies [8-12].

Infliximab, since its approval, is increasingly being used in patients with UC who are refractory to conventional therapies[13]. This treatment strategy aims at improving symptom control and reducing the need for hospitalization and surgery. Adalimumab is recommended for patients with Crohn's disease not responding to or unable to tolerate infliximab[14] due to its potential advantages over infliximab in terms of elimination of infusion reactions and possible reduction in the requirement for dose escalation over time [15]. However, the evidence on clinical performance of adalimumab over infliximab in UC is rather limited [16,17].

Despite the clinical benefits, induction and maintenance therapy using anti-TNF-α drugs is expensive. The estimated patient annual drug costs range from CA$23,000 to CA$38, 000 [18]. The prevalence of UC in Canada is 193.7 per 100,000 with 11.8 new cases per 100,000 each year [19]. Assuming 5% of the cases have moderate-to-severe refractory UC [20], approximately 8,500 patients will require treatment with an anti-TNF-α drug in Canada. Consequently, the volume of reimbursement requests, to the publicly funded drug plans in Canada, for infliximab and adalimumab from patients with refractory UC is increasing. These anti-TNF-α drugs are not listed as a general benefit in several provinces therefore reimbursement may be based on pre-set criteria or case-by-case reviews. There is a need in these jurisdictions for improving this process and developing reimbursement criteria or updating existing criteria. Therefore, this study was to evaluate the cost-utility of infliximab and adalimumab for the treatment of moderate-to-severe UC refractory to conventional therapies in Canada.

## Methods

### Study design

This was an economic evaluation of different medical management strategies for moderate-to-severe refractory UC from the perspective of a publicly funded healthcare system over a five-year time horizon. The relatively short time horizon was selected due to the lack of long-term clinical evidence of these drugs in UC. The target population was a hypothetical cohort of patients (aged 40 years and weighing 80 kg) with moderate-to-severe active refractory UC.

### Management strategies to be compared

**Figure 1:**
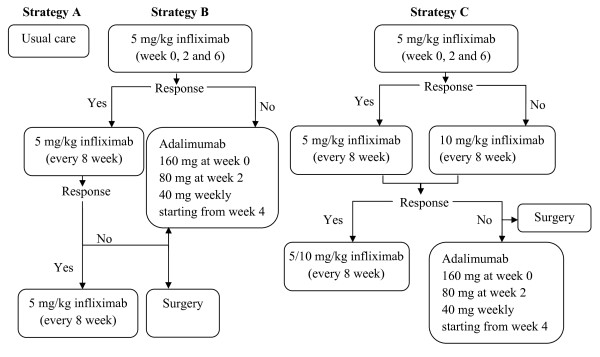
**Treatment strategies**.

The management strategies included (A) 'usual care': conventional medical treatment without anti-TNF-α drugs, the medications selected depended on the health states according to the inputs of two practicing gastroenterologists from tertiary care hospitals in two large urban regions within Ontario. There are four 5-ASA drugs commonly used for therapy in IBD. They suggested the proportion of patients utilizing each of the four 5-ASA drugs and the three immunosuppressant drugs based upon their own experiences (Table 1); (B) '5 mg/kg infliximab + adalimumab' initial and maintenance therapy using 5 mg/kg infliximab and then switch to adalimumab if there is no response to the initial therapy or response is lost during maintenance therapy; (C) '5 mg/kg and 10 mg/kg infliximab + adalimumab': initial therapy using 5 mg/kg infliximab, if there is no response, dose escalated to 10 mg/kg infliximab, and if response is lost during maintenance therapy, switch to adalimumab. Details on strategy B and C are shown in Figure 1.

**Table 1:** Profiles of concomitant medications and costs (2008 CA$)

Health state	Medications	Proportion	Daily dose	Drug costs per 12 weeks	Total costs of health state
Remission	None	-	-	-	$0.00
Active UC					
Responsive	5-ASA	0.9	4 g	$348.83	$698.44
	mesalamine enema	1.0	4 g	$384.50	
Nonresponsive	5-ASA	0.9	4 g	$348.83	$786.55
	mesalamine enema	1.0	4 g	$384.50	
	azathiopirne/6 MP*	0.4	150/75 mg	$222.76	

5-ASA: 5-aminosalicylates with an assumption of a 45:25:15:15 split of Pentasa:Asacol: salofalk:sulfasalazine.

*assumes 70:30 split of azathioprine:6 MP.

### Markov model

A five-health state Markov model was constructed to compare the incremental cost-utility of these three management strategies. The Active Ulcerative Colitis Trials 1 and 2 (ACT 1 and ACT 2) were the main sources of the clinical parameters used in the model[10]. The length of a Markov cycle was determined by the treatment intervals adopted in the ACTstudies.

**Figure 2:**
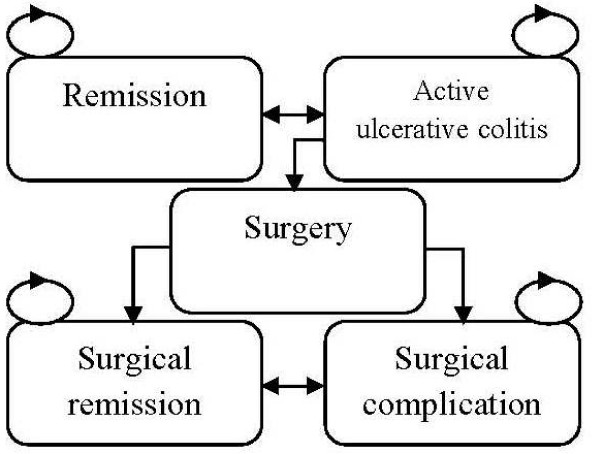
**Simplified Markov model schematic**.

Specifically, the first cycle was from week 0 to week 8, the second from week 9 to week 30, the third from week 31 to week 54, and then every 27 weeks for each cycle starting from week 55. Remission was defined as a total Mayo score of 2 points or lower, without individual sub-scores exceeding 1 point[10]. As the definitions of remission and response were overlapping in the ACT studies[10], responders defined in the present study excluded those achieving remission in order to make these two states mutually exclusive. The state of active UC consisted of both responders and non-responders to anti-TNF-α drugs. For those patients with remission after the initial therapy, they might lose response and thus move to active UC state, or stay in the remission state over time. For those responders in active UC, they might achieve remission or stay in the active UC state with the maintenance therapy. For those non-responders in the active UC state, a proportion of them underwent total colectomy with a one-stage ileal-pouch anal anastomosis (IPAA)[21] and the remaining either switched to adalimumab (as in strategy B) or increased the dose of infliximab (as in strategy C). The patients who experienced complications either achieved remission due to successful treatments for the complications (either medical or surgical), or still suffered complications despite the treatments. Those who achieved surgical remission might either develop complications later or stay in the remission state. Mortality was not considered due to the short time horizon. As the mortality rate is assumed the same for all strategies, impact of excluding mortality in the present evaluation would be minimal. A simplified schematic is displayed in Figure 2.

### Clinical parameters

Clinical parameters were estimated using a fixed-effect meta-analysis of the findings from the ACT studies[10]. The remission rates among responders and non-responders by treatment options are listed in Table 2. The clinical parameters for 'usual care' (strategy A) were estimated using those observed in the placebo arm of the ACT studies where some concurrent treatment with conventional drugs was allowed [10]. However, resource utilization and costs for the 'usual care' were based on the inputs from local clinicians to reflect the disease management process in Canada. A proportion of non-responders to medical treatment underwent surgery. The rate of surgery, within one year after the initial therapy, was 0.291 [8], while the rate of late surgery [i.e., beyond one year] was 0.087[22]. According to a large study by Fazio et al[23], the complication rate, within 30 days after the surgery, was 0.274 and the later complication rate (> 30 days) was 0.507. The overall success rate for the treatment of complications was 0.84[24]. These rates were not varied across treatment strategies. Table 3 shows time-dependent clinical parameters used in the model, including remission rate for each of the treatment options, the rates of maintaining remission over time and the proportion of non-responders in those with active UC. All these parameters were directly derived from the ACT studies[10]. To date only two small studies reported that adalimumab offered clinical benefits to a subgroup of patients (40% to 60%) that had a loss of response or were intolerant to infliximab[16,17]. Based on these findings, a conservative assumption was made that remission and response rates of maintenance therapy using adalimumab were equivalent to that of 5 mg/kg infliximab. The remission rate induced by adalimumab among those with no response or lost response to infliximab was 0.1 [16].

**Table 2:** Time-independent parameters used in the model

	Point estimate	SE	Distribution	**Ref**.
**Remission rates in responders**				
Usual care	0.146	0.047	Beta (α = 8.11; β = 47.34)	(10)
5 mg/kg infliximab	0.276	0.052	Beta (α = 20.20; β = 52.95)	(10)
10 mg/kg infliximab	0.255	0.047	Beta (α = 21.71; β = 63.44)	(10)
Adalimumab	0.276	0.052	Beta (α = 20.20; β = 52.95)	
**Remission rates in** **non-responders**				
Usual care	0.063	0.019	Beta (α = 10.17; β = 152.17)	(10)
10 mg/kg infliximab	0.078	0.029	Beta (α = 6.54; β = 76.96)	(10)
Adalimumab	0.100	0.020	Beta (α = 22.40; β = 201.60)	(16)
**Probabilities**				
Early IPAA	0.291	0.093	Beta (α = 6.71; β = 16.29)	(8)
Late IPAA	0.087	0.010	Beta (α = 66.99; β = 703.01)	(22)
Early complications	0.274	0.014	Beta (α = 275.65; β = 728.83)	(23)
Late complications	0.507	0.016	Beta (α = 527.02; β = 512.01)	(23)
Successful treatment for complications	0.840	0.031	Beta (α = 117.60; β = 22.40)	(24)
**Costs (2008CA$)**				
Infliximab, 100 mg/vial	952	50	Gamma(α = 362.44; β = 2.63)	(25,26)
Adalimumab, 400 mg	715	46	Gamma(α = 237.56; β = 2.98)	(25,26)
Medical examination	77			(27)
IPAA	12,738	2548	Gamma(α = 25.00; β = 509.52)	(28)
Surgical complications	9304	2301	Gamma(α = 16.35; β = 569.08)	(28)
**Utilities**				
Remission	0.79	0.035	Beta (α = 108.43; β = 28.82)	(21)
Active UC	0.32	0.045	Beta (α = 34.46; β = 73.23)	(21)
Surgical remission	0.68	0.042	Beta (α = 83.77; β = 39.42)	(21)
Surgical complications	0.49	0.046	Beta (α = 56.91; β = 59.23)	(21)

SE: standard error; IPAA: one-stage ileal-pouch anal anastomosis; UC: ulcerative colitis.

**Table 3:** Time-dependent parameters used in the model*

	Point estimate (standard error)			
Time	Usual care	5 mg/kg infliximab	10 mg/kg infliximab	Adalimumab
***Week 0-8***				
Remission rate	0.084(0.017)	0.363(0.031)	0.297(0.029)	0.363(0.031)
% Non-responders among patients with active UC	74.4(2.9)	51.9(4.0)	49.4(3.8)	51.9(4.0)
				
***Week 9-30***				
Maintaining remission rate	0.520(0.099)	0.524(0.053)	0.820(0.045)	0.524(0.053)
% Non-responders among patients with active UC	83.0(2.6)	71.8(3.5)	71.3(3.6)	71.8(3.5)
				
***Week 31-54***				
Maintaining remission rate	0.799(0.127)	0.856(0.066)	0.781(0.073)	0.856(0.066)
% Non-responders among patients with active UC	96.0(1.9)	83.5(4.2)	85.0(4.0)	83.5(4.2)
				
***Week>54***				
Maintaining remission rate	0.799(0.127)	0.856(0.066)	0.781(0.073)	0.856(0.066)
% Non-responders among patients with active UC	96.0(1.9)	83.5(4.2)	85.0(4.0)	83.5(4.2)

Active UC refers to active ulcerative colitis that consists of both responders and non-responders.

*The parameters were derived from the study by Rutgeerts et al.[10].

### Costs and utilities

The cost of infliximab was $952 per 100 mg/vial and the cost of adalimumab was $715 per 40 mg according to the provincial drug benefit lists[25,26]. An 8% markup fee was added to the drug costs. Total costs of medical examination including chest x-ray, tuberculosis skin test, and hepatitis B blood test were estimated at $77 according to the Ontario Schedule of Benefits[27]. Cost of IPAA was estimated at $12,738 [28]. All costs were reported in 2008 Canadian dollars. Utilities for the four Markov health states (surgery was a temporary transition state only) were obtained from the study by Arseneau et al[21] (Table 2). Both costs and quality-adjusted life years (QALYs) were discounted at 5% annually in the base case analysis, while 0% and 3% were used in sensitivity analysis.

### Outcomes

The primary outcome measure was the incremental cost-utility ratio (ICUR) between the strategies with 'usual care' as a reference group.

### Base case and sensitivity analyses

The base case analysis estimated the ICURs between the strategies using point estimates of the parameters (i.e., weighted-average from the meta-analysis). One-way deterministic sensitivity analyses (DSA) were performed using the lower and upper bounds of the 95% confidence interval of the parameters that were assumed constant over time. Probabilistic sensitivity analysis (PSA) was conducted using Monte Carlo simulations. Beta distributions (generating values bounded between 0 and 1) were fitted for the clinical parameters and utilities, while gamma distributions (generating positive values) were fitted for the cost parameters. Cost-effectiveness acceptability curves (CEACs) were constructed to show the varying probabilities of being the optimal strategy at different willingness to pay (WTP) threshold values.

## Results

In the base case analysis, strategy A ('usual care') cost $24,268 and yielded 2.015 QALYs for a patient over a 5-year period. The corresponding numbers were $82,756 and 2.178 QALYs for strategy B ('5 mg/kg infliximab + adalimumab') and $101,272 and 2.149 QALYs for strategy C ('5 mg/kg and 10 mg/kg infliximab + adalimumab'). The ICUR was $358,088/QALY for strategy B versus 'usual care' and $575,540/QALY for strategy C versus usual care. Strategy C was dominated by strategy B (Table 4).

**Table 4:** Base case and one-way deterministic sensitivity analyses

		ΔCosts		ΔQALYs		ICURs, $/QALY	
	Values	B vs A	C vs A	B vs A	C vs A	B vs A	C vs A
**Base case**		$58,488	$77,004	$0.163	$0.134	$358,088	$575,540
							
**Remission rate in responders**							
Usual care	0.054	$58,049	$76,557	0.181	0.151	$320,893	$505,803
	0.238	$58,937	$77,446	0.146	0.117	$402,860	$663,307
5 mg/kg infliximab	0.174	$57,360	$76,880	0.121	0.100	$476,059	$768,537
	0.378	$59,592	$77,143	0.203	0.166	$293,330	$464,724
							
**Remission rate in non-responders induced**							
Adalimumab	0.061	$58,054	$76,876	0.123	0.120	$471,182	$641,097
	0.139	$58,923	$77,133	0.200	0.146	$295,032	$526,848
**Early IPAA**	0.108	$72,853	$95,814	0.254	0.215	$286,943	$446,029
	0.475	$47,476	$61,931	0.104	0.082	$457,710	$753,220
**Utilities**							
Remission	0.720	$58,499	$77,007	0.111	0.087	$527,236	$889,227
	0.858	$58,499	$77,007	0.214	0.180	$273,081	$428,676
Surgical remission	0.598	$58,499	$77,007	0.197	0.167	$296,939	$462,473
	0.762	$58,499	$77,007	0.130	0.101	$451,163	$761,873
Surgical complications	0.399	$58,499	$77,007	0.185	0.155	$316,155	$497,122
	0.580	$58,499	$77,007	0.142	0.113	$412,327	$681,998

Strategy A: 'usual care'; Strategy B: '5 mg/kg infliximab +adalimumab'; Strategy C: '5 mg/kg and 10 mg/kg infliximab + adalimumab'. IPAA: one-stage ileal-pouch anal anastomosis; UC: ulcerative colitis.

The ICURs did not vary greatly when using 0% and 3% discount rates. Those parameters showing a significant impact on the ICURs in a one-way DSA are displayed in Table 4. The ICURs were sensitive to the remission rates maintained by 'usual care' and 5 mg/kg infliximab, and also sensitive to the rate of remission induced by adalimumab in non-responders to infliximab. Early IPAA rate and utility values had a significant impact on the ICURs, while the impacts from the remaining parameters were negligible.

**Figure 3:**
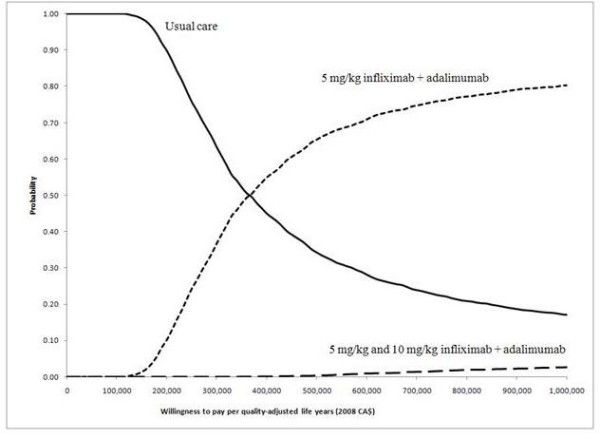
**Cost-effectiveness acceptability curves**.

CEACs are shown in Figure 3. When the WTP was less than $150,000/QALY, the probability of 'usual care' being the optimal strategy was 1.0. The probability of strategy B being optimal was 0.5 when the WTP approximated $400,000/QALY. The probability of strategy C being optimal was very low despite the wide range of WTP values.

## Discussion

Increasing health care costs with limited resources is highlighting the importance of economic issues in addition to efficacy and safety. In Canada, economic evaluation has been incorporated into the decision making process since 1994. Facing increasing volume of reimbursement requests for anti-TNF-α drugs, the Canadian decision makers have to evaluate these drugs in terms of incremental cost-utility over the existing treatment options.

Infliximab has demonstrated clinical efficacy in patients with active UC in randomized clinical trials[10,16]. However, the costs of infliximab were significantly higher than the costs of 'usual care'. The ICUR was not favorable to infliximab based on the existing evidence, according to commonly accepted willingness to pay threshold (e.g., US $50,000).

This study demonstrated that increasing the dose of infliximab to 10 mg/kg in those patients who had a loss of response to 5 mg/kg infliximab was not cost-effective compared to either 'usual care' or switching to adalimumab. Clinical experts also suggested a strategy of reducing the dose interval for non-responders. However, this strategy was not considered in the model due to lack of clinical evidence.

Adalimumab also induced remission among a certain proportion of the patients intolerant or unresponsive to infliximab therapy[16,17]. The performance of adalimumab in those without a response to infliximab has not been extensively studied to date. Only two small studies from the same research group have been published[16,17]. The long-term performance of adalimumab remains unclear. A large randomized controlled trial is necessary to address this concern. Nevertheless, the use of infliximab and adalimumab in patients who are refractory to conventional therapies might be clinically indicated, while the economic burden of these treatments should be taken into consideration.

Also, there is a paucity of economic evidence on anti-TNF-α drugs in treating refractory active UC. A recently published economic evaluation of infliximab in the UK reported an ICUR of £27,424 if adopting a responder strategy (i.e., continuing 5 mg/kg infliximab in responders only), while the ICUR was £19,696 for the remission strategy (i.e., continuing 5 mg/kg infliximab in patients achieving remission only), compared to 'usual care' comprising immunomodulators or corticosteroids[29]. These ICURs were significantly lower than those in the present study. Although using the same data source (i.e., ACT studies), the two studies were less comparable in terms of model structure, model assumption, and management strategies. Specifically, the UK model had a state for temporary discontinuers. The target population in the UK study was the patients with moderate-to-severe UC, while our population was those with similar severity but refractory to conventional treatment. In the UK study patients that did not respond to infliximab were allowed to switch back to conventional treatment.

The present model did not incorporate adverse events as the ACT studies reported that the proportions of patients with any adverse event were similar among the three treatment groups. Nevertheless, the ICUR would be slightly favorable to 'usual care' if adverse events were included in the model. For example, the numbers of serious infections (i.e. lupus-like reactions and neurologic diseases) were slightly higher among patients treated with infliximab in the ACT studies. Incorporating these infections into the model would increase costs and decrease health benefits in terms of utility for the treatment strategies using infliximab. The present model was also limited by not incorporating subsequent post surgery treatment strategies. This is mainly because these treatments are individualized and thus hard to estimate in a cohort model. This could be a clinical research priority in the future.

This economic evaluation revealed that the ICURs of anti-TNF-α drugs were not satisfactory in treating patients with moderate-to-severe refractory UC in a relatively short treatment period. Future research could be aimed at the long-term clinical benefits of these drugs, especially adalimumab for patients intolerant or unresponsive to infliximab treatment.

## Competing interests

FX, NA, and DR disclosed no conflicts of interest. GB received funding from Eli Lilly Canada Inc. and GlaxoSmithKline Inc. for consulting. KG received funding grants from Abbott Laboratories Ltd. for a speaking engagement. RG was advisory board member to Janssen-Ortho Inc. and Hoffman-La Roche Ltd. and consultant for Eli Lilly Canada Inc.

## Authors' contributions

FX participated in the conception and design of the review, acquired the data, performed the analysis and interpretation of the data and drafted the article. GB participated in the conception and design of the review and revised the article for critically important intellectual content. NA participated in acquiring the data and revised the article for critically important intellectual content. KG participated in acquiring the data and revised the article for critically important intellectual content. DR participated in acquiring the data and revised the article for critically important intellectual content. RG participated in the conception and design of the review and revised the article for critically important intellectual content. All authors read and approved the final manuscript.
